# Stoichiometry and intracellular fate of TRIM-containing TCR complexes

**DOI:** 10.1186/1478-811X-8-5

**Published:** 2010-03-18

**Authors:** Mahima Swamy, Gabrielle M Siegers, Gina J Fiala, Eszter Molnar, Elaine P Dopfer, Paul Fisch, Burkhart Schraven, Wolfgang WA Schamel

**Affiliations:** 1 Department of Molecular Immunology, Max Planck-Institute of Immunobiology and Institute for Biology III, Albert Ludwigs University Freiburg, Stübeweg 51, 79108 Freiburg, Germany; 2 Cell Therapy Program, Princess Margaret Hospital/Ontario Cancer Institute, 610 University Ave., Toronto, Ontario, M5G 2M9, Canada; 3 Spemann Graduate School of Biology and Medicine (SGBM), Albert Ludwigs University Freiburg, Albertstraße 19A, 79104 Freiburg, Germany; 4 Department of Pathology, University of Freiburg Medical Center, 79110 Freiburg, Germany; 5 Institute of Molecular and Clinical Immunology, Otto-von-Guericke-Universität Magdeburg, Leipziger Strasse 44, 39120 Magdeburg, Germany

## Abstract

**Background:**

Studying the stoichiometry and intracellular trafficking of the T cell antigen receptor (TCR) is pivotal in understanding its mechanisms of activation. The αβTCR includes the antigen-binding TCRαβ heterodimer as well as the signal transducing CD3εγ, CD3εδ and ζ_2 _subunits. Although the TCR-interacting molecule (TRIM) is also part of the αβTCR complex, it has not been included in most reports so far.

**Results:**

We used the native antibody-based mobility shift (NAMOS) assay in a first dimension (1D) blue native (BN)-PAGE and a 2D BN-/BN-PAGE to demonstrate that the stoichiometry of the digitonin-solublized TRIM-containing αβTCR is TCRαβCD3ε_2_γδζ_2_TRIM_2_. Smaller αβTCR complexes possess a TCRαβ CD3ε_2_γδζ_2 _stoichiometry. Complexes of these sizes were detected in T cell lines as well as in primary human and mouse T cells. Stimulating the αβTCR with anti-CD3 antibodies, we demonstrate by confocal laser scanning microscopy that CD3ε colocalizes with ζ and both are degraded upon prolonged stimulation, possibly within the lysosomal compartment. In contrast, a substantial fraction of TRIM does not colocalize with ζ. Furthermore, TRIM neither moves to lysosomes nor is degraded. Immunoprecipitation studies and BN-PAGE indicate that TRIM also associates with the γδTCR.

**Conclusions:**

Small αβTCR complexes have a TCRαβ CD3ε_2_γδζ_2 _stoichiometry; whereas those associated with one TRIM dimer are TCRαβ CD3ε_2_γδζ_2_TRIM_2_. TRIM is differentially processed compared to CD3 and ζ subunits after T cell activation and is not degraded. The γδTCR also associates with TRIM.

## Background

T cells are classified as αβ or γδ T cells depending on the type of T cell receptor (TCR) that they express. The γδTCR is expressed on γδ T cells and is involved in their development and activation. γδTCR ligands are largely undefined, but include protein and non-protein substances [1,2]. The αβTCR controls the development and activation of αβ T cells, and thus plays a critical role in the adaptive immune system. Its ligands are non-self peptides presented by major histocompatibility complex molecules (MHC) on the surface of antigen presenting cells (APC).

The αβTCR consists of TCRαβ, CD3γε, CD3δε and ζζ (CD247_2_) dimers that are non-covalently bound to one another [3,4]. The heterodimeric TCRαβ contains variable immunoglobulin (Ig) domains, which bind to peptide/MHC-complexes. Furthermore, it possesses a transmembrane (TM) region that mediates its association with the CD3 subunits via potentially positively charged amino acids [5-7]. Each CD3 subunit contains an invariable extracellular part, a TM region carrying potentially negatively charged amino acids and a cytoplasmic tail with several signal transduction motifs, including an immunoreceptor tyrosine-based activation motif (ITAM) [8]. The ζ chain contains only 9 extracellular amino acids buried within the αβTCR complex [9,10], a TM region with a potentially negatively charged amino acid that also binds to TCRαβ, and a cytoplasmic tail with three ITAMs. Upon ligand binding to TCRαβ, the CD3 subunits undergo a conformational change [11-14] and the ITAMs within the CD3 cytoplasmic tails and ζ-chains become phosphorylated by protein tyrosine kinases of the Src-family. They subsequently recruit SH2-domain containing intracellular signaling and adapter molecules to the plasma membrane. Hence, while the TCRαβ heterodimer is responsible for binding of the antigen, the CD3 and ζ subunits of the αβTCR serve as signal transducing elements. The γδTCR is composed of the same signal transducing elements while carrying the antigen binding TCRγδ heterodimer.

In addition to the above molecules, the TCR complex also includes the transmembrane adapter protein TRIM (TCR-Interacting Molecule) [15-17]. The association of TRIM with the αβTCR is suggested by the following observations: (i) TRIM co-purifies either with the endogenous αβTCR [16,18] or individually expressed ζ [18] under mild detergent conditions, (ii) TRIM co-caps with CD3ε upon antibody-mediated TCR-crosslinking in αβ T cells [18] and (iii) overexpression of TRIM leads to enhanced αβTCR expression at the cell surface [18]. However, in contrast to CD3 and ζ, TRIM is not mandatory for αβTCR assembly and surface expression [19-22].

TRIM is structurally similar to the ζ chain; it has a short extracellular domain, a long cytoplasmic tail and is a disulfide-linked homodimer. However, its cytoplasmic tyrosine residues are not contained within typical ITAM sequences, but rather within YxxM-motifs that might mediate binding of the p85 subunit of PI3K [16]. TRIM is phosphorylated upon TCR-stimulation by Src protein tyrosine kinases [16] and, together with other transmembrane adapter proteins (e.g. SIT), modulates TCR-mediated signals thereby regulating thymocyte development [22,23]. The precise signaling function of TRIM remains elusive, since the TRIM knock-out mouse did not show any phenotype in the assays employed [22].

Using the native antibody-based mobility shift (NAMOS) assay, we recently revealed the stoichiometry of the basic αβTCR complex extracted from T cell membranes which is TCRαβ CD3ε_2_γδζ_2 _[24-27]. The stoichiometry of the γδTCR is TCRγδ CD3ε_2_γδζ_2 _(human) and TCRγδ CD3ε_2γ2ζ2 _(mouse) [28]. However, in these studies we did not look for TRIM-containing TCR complexes. Therefore, the present study was aimed at deciphering the stoichiometry of TRIM-containing TCRs. Furthermore, we analyzed the intracellular fate of TRIM upon TCR-stimulation in comparison with that of other components of the TCR complex.

## Results

### The αβTCR forms two complexes, one of which contains TRIM

**Figure 1:**
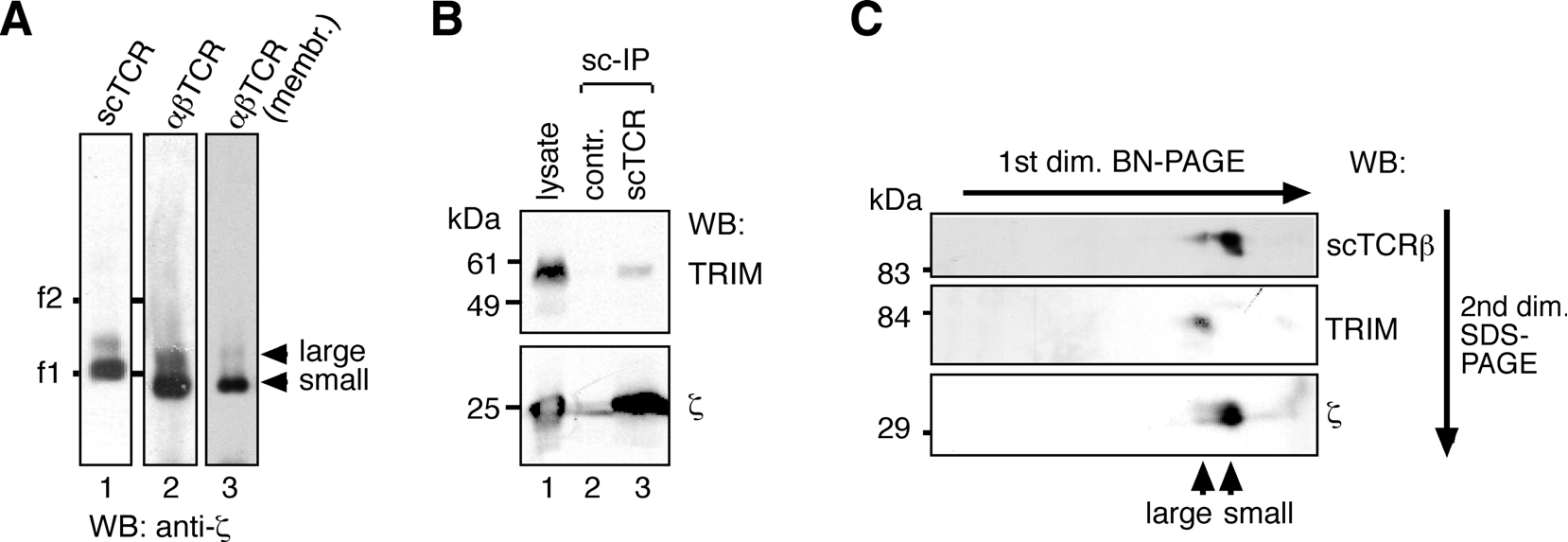
**The large αβTCR complex contains TRIM**. (A) The single chain-tagged TCR (scTCR) was purified from digitonin-lysed 31.13scTCRβ cells using NP-sepharose affinity columns and free NIP for elution (lane 1). Pervanadate-stimulated Jurkat cells were lysed in digitonin and αβTCRs purified with anti-phosphotyrosine antibodies followed by phenylphosphate elution (lane 2). Membranes from Jurkat cells were lysed in digitonin (lane 3). TCRs were separated by BN-PAGE and immunoblotted against ζ. The marker protein was ferritin in its 24- and 48-meric forms (f1: 440 kDa, f2: 880 kDa). (B) 31.13scTCRβ (lanes 1 and 3) and Jurkat (lane 2) cell lysates were subjected to affinity purification on NP-sepharose columns. The purified proteins were separated on a reducing SDS-PAGE; TRIM and ζ were detected by immunoblotting. (C) NP-purified scTCRs were separated by 1D BN-PAGE and 2D non-reducing SDS-PAGE. Immunoblotting was done against scTCRβ, TRIM and ζ.

The TCR complex of the TCRαβ CD3ε_2_γδζ_2 _stoichiometry resolves in BN-PAGE just below the f1 marker [24-27]. We noticed that digitonin-purified human TCRs often showed a second larger complex in BN-PAGE [25]. To characterize this complex biochemically, using digitonin we purified the αβTCR from the 31.13scTCRβ T cell line [25], which expresses a single chain-tagged TCRβ subunit (scTCRβ) [11]. The sc-tag binds nitrophenol (NP) with moderate and nitroiodophenol (NIP) with high affinity [29]. By binding to NP-coupled columns and subsequently eluting the bound complexes with free NIP, we purified the scTCR in its native form. After separation by BN-PAGE, the eluted αβTCR complexes were detected with an anti-ζ antiserum. Figure 1A, lane 1 shows that under these experimental conditions two distinct αβTCRs were detected. Note that the αβTCR from Jurkat cells showed the same two bands, although smaller in size due to the absence of the sc-tag (lanes 2 and 3). The larger complex was stimulation-independent, since it was present in pervanadate-stimulated (lane 2) as well as in unstimulated (lane 3) Jurkat cells. The increase in molecular weight for the larger complexes indicated an additional mass of 50 to 100 kDa (not shown).

TRIM forms disulfide-linked dimers of 60 kDa and noncovalently associates with the TCR [16,18]. To test whether TRIM is associated with the scαβTCR, we lysed 31.13scTCRβ or Jurkat cells in digitonin and affinity-purified αβTCR complexes with NP-sepharose. The purified material was separated on a reducing SDS-PAGE. TRIM and ζ were detected by Western blotting. The untagged αβTCR from Jurkat cells did not bind to NP-sepharose and thus TRIM was not detected (lane 2); however, both TRIM and ζ were purified from scTCR lysates, (Figure 1B, lane 3), indicating that TRIM associated to the scTCR. This is in line with the binding of TRIM to αβTCRs [16,18].

Next we tested whether TRIM was part of the larger αβTCR complex seen in BN-PAGE. To this end, the two scTCR complexes in the BN-PAGE were further separated by a second dimension non-reducing SDS-PAGE and analysed by anti-TCRβ and anti-ζ Western blotting. This approach revealed that both small and large αβTCR-complexes contained TCRβ and ζ (Figure 1C, upper and lower panel). The signal detected by anti-TCRβ was at 90 kDa and that detected by anti-ζ was at 35 kDa, corresponding to the TCRαβ and ζζ dimers, respectively. Immunoblotting against TRIM revealed that TRIM dimers of 50-60 kDa were solely present in the larger αβTCR complex. Thus, two αβTCR complexes exist in digitonin lysates, one with TRIM and one without.

### Stoichiometry of TRIM-containing αβTCR complexes

**Figure 2:**
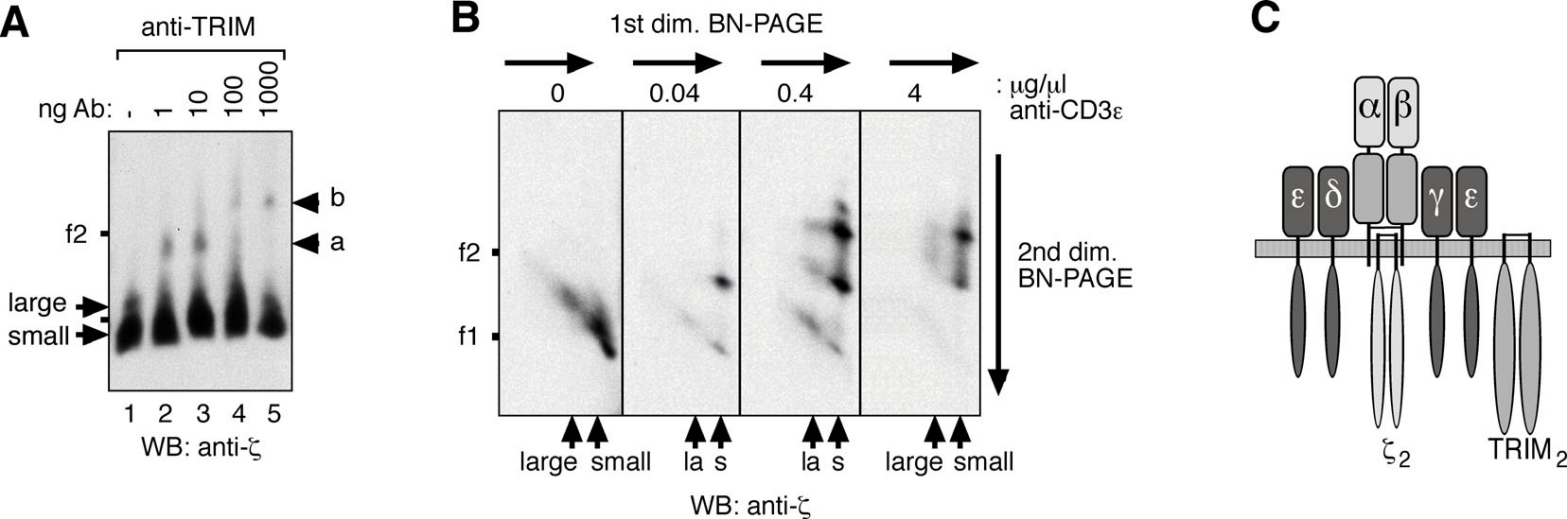
**The stoichiometry of the TRIM-containing αβTCR**. (A) Digitonin-solubilized scTCRs from 31.13scTCRβ cells were purified from NP-sepharose affinity columns using free NIP for elution. Before loading on a native gel, the TCR was incubated with the indicated amounts of the anti-TRIM antibody TRIM-7, and after separation, immunoblotted with anti-ζ. αβTCR complexes bound to one or two antibodies are marked with a or b, respectively. Ferritin was used as a marker protein as in figure 1. (B) αβTCRs were purified as in (A) and after separation by a 1D BN-PAGE, each lane was subjected to a 2D BN-PAGE into which the indicated amounts of the anti-CD3ε antibody UCHT1 had been loaded. The anti-ζ antiserum was used for Western blot detection. Arrows: small, the TCRαβ CD3ε_2_γδζ_2 _TCR; and large, the TCRαβ CD3ε_2_γδζ_2_TRIM_2 _TCR. (C) a schematic of the TRIM-containing large αβTCR complex is shown.

The NAMOS assay can be used to assess stoichiometries of multiprotein complexes [25,26]. When the NAMOS assay was performed using anti-TRIM antibodies, we found that part of the αβTCR shifted once at low antibody concentrations (Figure 2A, band a), indicating one antibody bound to the αβTCR. With high amounts of anti-TRIM antibody, a second shift was generated (band b) that likely contains two anti-TRIM antibodies. Therefore, two anti-TRIM binding sites and thus two copies of the TRIM protein are present, indicating that one TRIM homodimer [16] is a component of the large αβTCR complex.

Next, we modified the NAMOS-assay, using it in a second dimension (2D) BN-PAGE to also assess the amount of CD3ε within the complex. The presence of two αβTCR complexes precludes the use of the NAMOS-assay in a 1D BN-PAGE, since both large and small αβTCR complexes would shift, resulting in several bands, which would be difficult to assign. We separated the two αβTCRs by BN-PAGE, and subsequently let antibody bind during a further separation by 2D BN-PAGE. We tested various experimental conditions to bring the antibody into contact with the complexes inside the first dimension BN-PAGE (not shown). The protocol that worked best was to load varying amounts of antibody onto the 2D gel and allow it to enter the stacking gel by brief electrophoresis. Subsequently, the 1D gel slices containing the αβTCR complexes were placed over the 2D stacking gel and electrophoresis was continued. When we applied this 2D BN-/BN-PAGE NAMOS-assay to the αβTCRs using an anti-CD3ε antibody, we detected two shifts for both complexes (Figure 2B), indicating that both contained two CD3ε subunits. A third shift evident at 0.4 μg/μl anti-CD3ε is attributed to two αβTCR complexes crosslinked by one antibody, a phenomenon occurring at non-saturating antibody concentrations (Figure 2B, 3rd panel). The large αβTCR complex thus contains TCRαβ (Figure 1C), ζ_2 _(Figures 1 and 2), one TRIM homodimer (Figure 2A) and two CD3ε (Figure 2B). Since CD3ε always pairs with CD3γ and CD3δ [3] it can be concluded that the TRIM-containing αβTCR has a stoichiometry of TCRαβ CD3ε_2_γδζ_2_TRIM_2 _(Figure 2C).

### TRIM is loosely associated with the αβTCR

**Figure 3:**
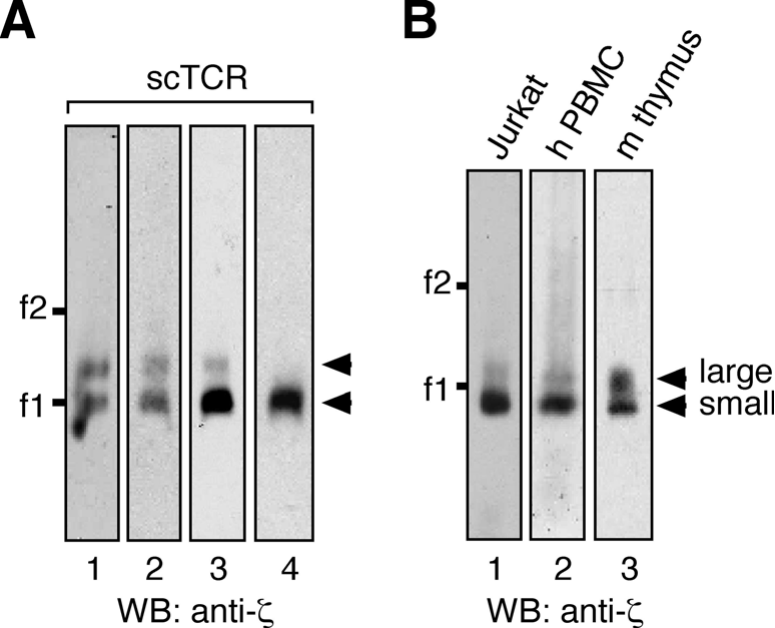
**Primary cells express the TRIM-containing αβTCR**. (A) 31.13scTCRβ cells were lysed with digitonin on different days using different lots of digitonin. scTCRs were purified from NP-sepharose affinity columns using free NIP for elution. TCRs were separated by BN-PAGE and immunoblotted against ζ. (B) TCRs were purified from pervanadate-stimulated Jurkat cells as in figure 1A (lane 1). Membranes were prepared from human PBMC and lysed in digitonin (lane 2). Murine thymocytes were stimulated with pervanadate and and TCRs purified with anti-phosphotyrosine antibodies followed by phenylphosphate elution (lane 3). TCRs were separated by BN-PAGE and immunoblotted against ζ. Ferritin was used as a marker protein as in figure 1.

We have observed the larger TRIM-containing αβTCR in BN-PAGE over many years. One example is shown in figure 3, where we have used the scTCR (Figure 3A). Depending on the experiment, the proportion of TRIM-containing αβTCRs can vary from 50% of all αβTCRs (lane 1) to undetectable amounts (lane 4). The reasons for this inconsistency might include: different lots of the detergent digitonin used for αβTCR extraction and washing, different detergent/lipid ratios and slightly different purification and separation conditions. The large TRIM-αβTCR band has a defined size, indicating that it contains a specific protein complex and is not an artefact caused by protein aggregation. The observation that TRIM is easily lost from the αβTCR complex shows that TRIM is loosely associated and that a large percentage of αβTCRs might contain TRIM in the living cell.

### The TRIM-containing αβTCR is present in primary T cells

To extend our studies to primary cells from human and mouse, we used human peripheral blood T lymphocytes as well as murine thymocytes (Figure 3B, lanes 2 and 3). Jurkat cells served as a control (lane 1). Digitonin-solubilized αβTCR complexes were separated by BN-PAGE. The larger αβTCR complex, that most likely includes TRIM, was present in primary cells. This is in line with our earlier data showing that TRIM co-caps with CD3 in primary human cells [18] and that TRIM is expressed in murine thymocytes [30].

### TRIM is also a component of the γδTCR complex

**Figure 4:**
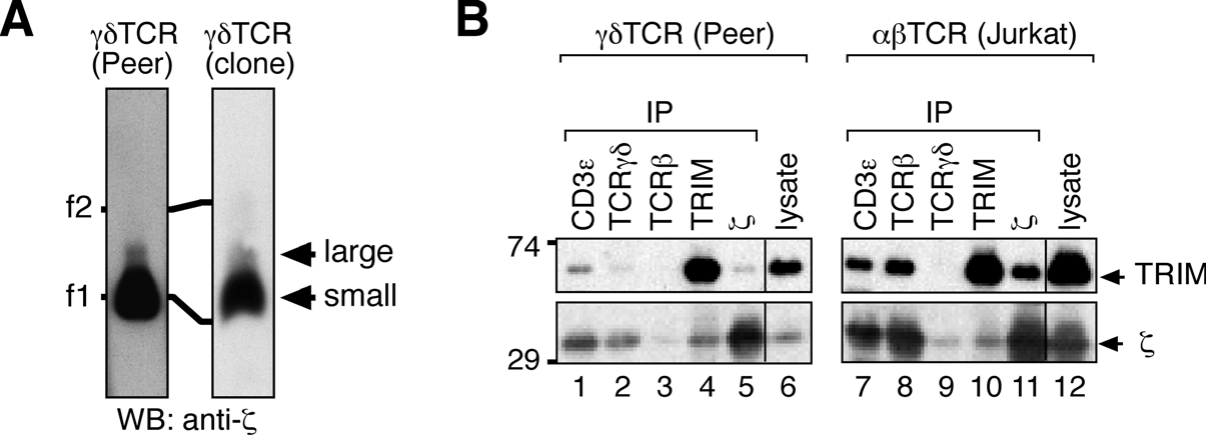
**TRIM is part of the γδTCR complex**. (A) The human γδ T cell line Peer (left) and a human Vγ9Vδ2 clone (right) were pervanadate-stimulated and lysed in digitonin. γδTCRs were purified with anti-phosphotyrosine antibodies followed by phenylphosphate elution. TCRs were separated by BN-PAGE and immunoblotted against ζ. Ferritin was used as a marker protein as in figure 1. (B) Peer γδ and Jurkat αβ T cells were lysed and immunoprecipitations performed with antibodies recognizing the indicated proteins (lanes 1 - 5 and 7 - 11). Purified proteins, as well as lysates (lanes 6 and 12), were separated by non-reducing SDS-PAGE. TRIM and ζ were detected by immunoblotting.

We are also interested in the γδTCR complex. We have shown that the most abundant human γδTCR after digitonin extraction has a stoichiometry of TCRγδ CD3ε_2_γδζ_2 _[28]. To test whether a second larger complex of the γδTCR is detected in BN-PAGE, we analyzed digitonin-purified γδTCRs from the human γδ T cell line Peer or from a human γδ T cell clone. Peer cells express a γδTCR containing the TCRγ2 protein and the clone a Vγ9Vδ2 receptor (data not shown) including the TCRγ1 protein. Separated γδTCRs were detected by Western blotting using anti-ζ antibodies (Figure 4A). Under these experimental conditions two distinct γδTCRs were detected. The size difference is likely due to the incorporation of one TRIM dimer in the larger complex. To confirm that TRIM binds the γδTCR, we immunopurified the γδTCR from lysates of Peer γδ T cells using anti-TCR specific antibodies. Complexes were separated by non-reducing SDS-PAGE; TRIM and ζ were detected by Western blotting (Figure 4B). In control samples, TRIM was absent in anti-TCRβ precipitates prepared from Peer cells (lane 3) and anti-TCRγδ precipitates from Jurkat cells (lane 9). However, TRIM was found in anti-CD3ε, anti-TCRγδ and anti-ζ immuno-isolates of Peer cells (lanes 1, 2 and 5) as well as anti-CD3ε, anti-TCRαβ and anti-ζ immuno-isolates of Jurkat T cells (lanes 7, 8 and 11). Thus, TRIM also associates with the γδTCR, independent of which TCRγ chain is used.

### Intracellular fate of TRIM after αβTCR stimulation

**Figure 5:**
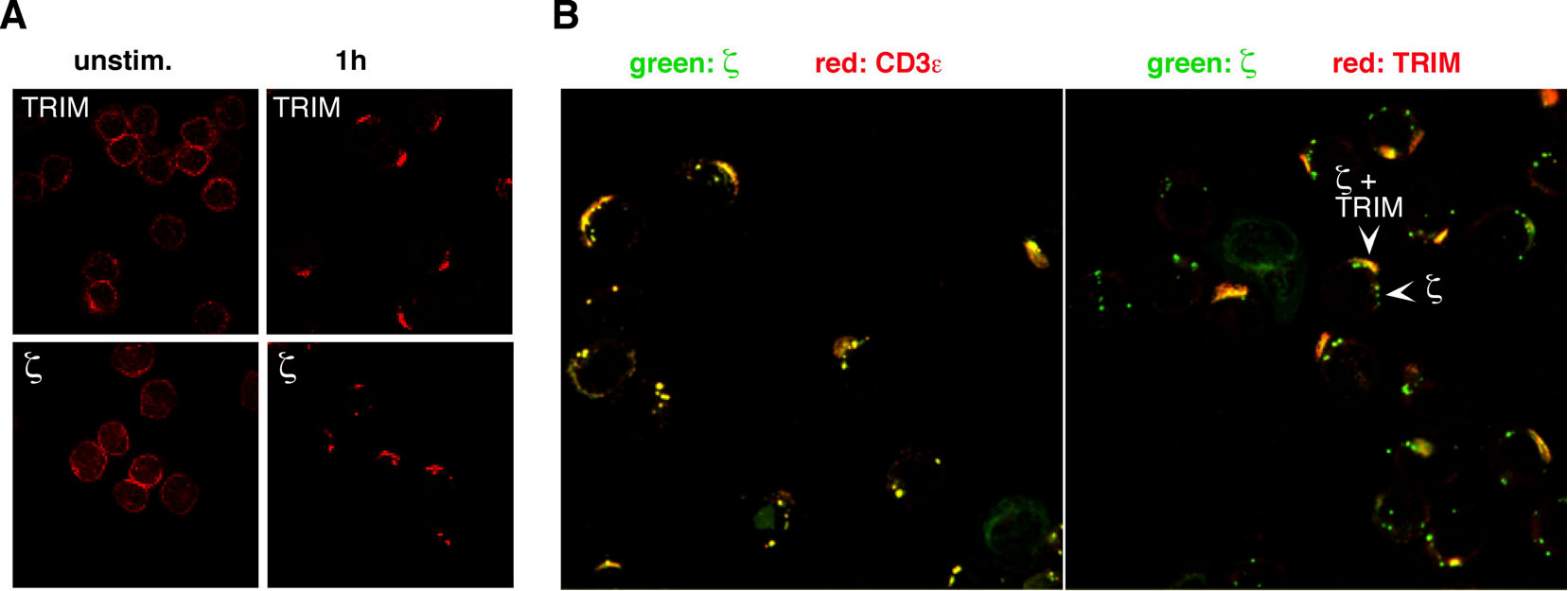
**Colocalization of TRIM with CD3ε and ζ 1 hour post-αβTCR activation**. (A) Human peripheral blood T cells were incubated with the IgM anti-CD3ε mAb 2Ad2a2 for 1 hour or left unstimulated. Cells were fixed and the subcellular localization of TRIM and ζ was determined using the antibodies TRIM-7 mAb (top) and anti-ζ mAb 6B10.2 (bottom). Secondary staining was done with a Texas red-labeled donkey anti-mouse antiserum. (B) Cells were stimulated as in (A), then fixed and the subcellular localization of ζ detected using FITC-labelled anti-ζ mAb 6B10.2. CD3ε was visualized using Texas Red-labelled goat anti-mouse IgM (left) and a biotinylated TRIM-4 mAb followed by Texas Red-coupled streptavidin were used to detect TRIM (right).

Upon binding of peptide/MHC-complexes the αβTCR is endocytosed and degraded in lysosomes [31]. To assess whether TRIM is also internalized and degraded upon T-cell activation, we stimulated human peripheral blood T lymphocytes with anti-CD3ε mAb and subsequently investigated the subcellular localization of TRIM by means of confocal laser scanning microscopy. As previously reported, TRIM showed strong capping at the T cell surface after one hour of αβTCR stimulation (Figure 5A, upper panel). Similarly, αβTCR stimulation induced capping of the ζ chain (lower panel).

**Figure 6:**
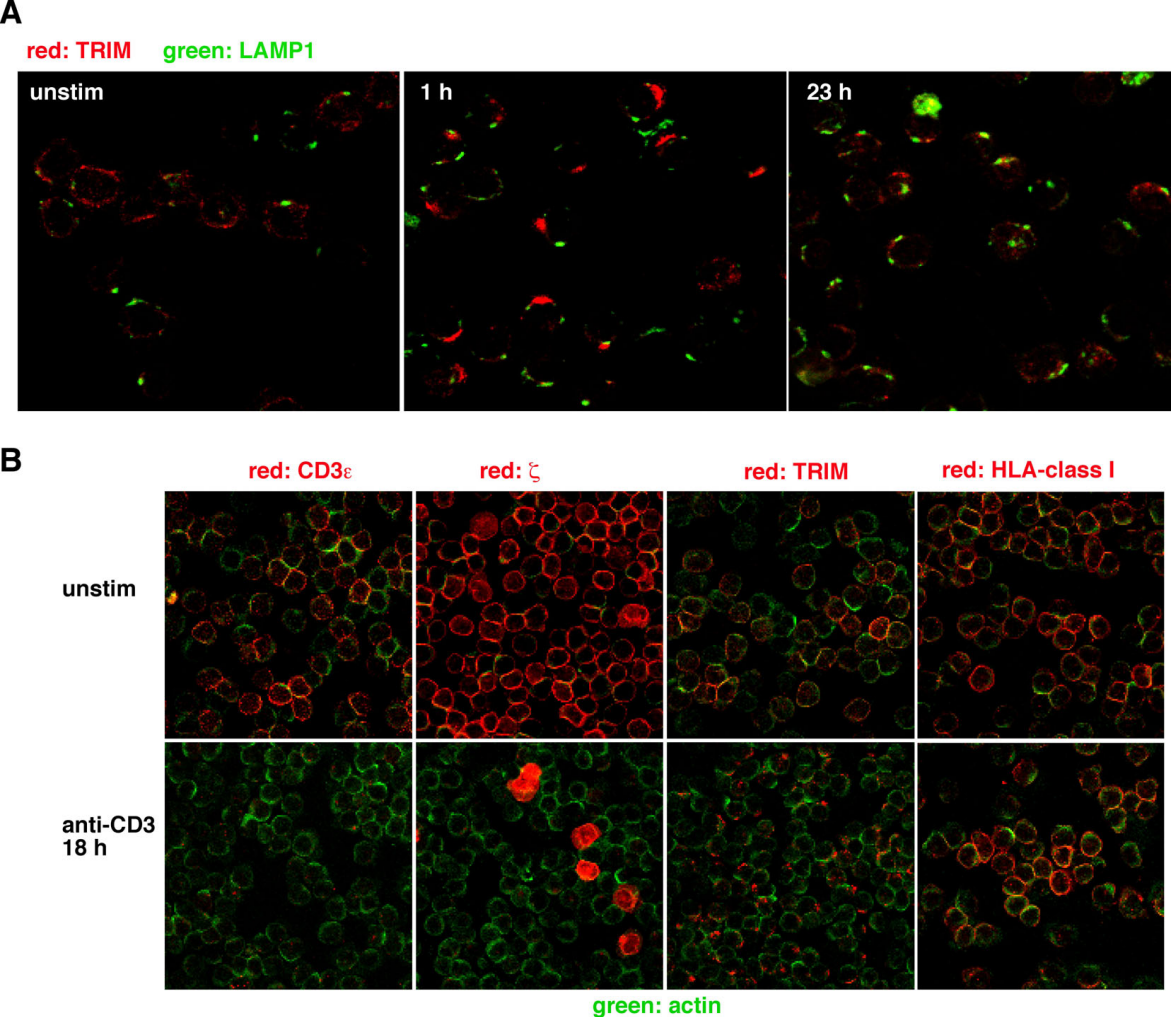
**Differential processing of TRIM compared to other αβTCR complex components**. (A) Human peripheral blood T cells were incubated with anti-CD3ε mAb 2Ad2a2 to induce cap formation for 1 and 23 hours or left unstimulated. Cells were fixed and the subcellular localization of TRIM detected using biotinylated TRIM-4 mAb and Texas Red-coupled streptavidin; LAMP1 was stained with FITC-labelled anti-Lamp 1 antibody. (B) Cells were unstimulated (top) or stimulated as above for 18 hours. In all panels, actin was visualized using a Cy2-labelled goat anti-rabbit antiserum. Primary mAb antibodies were: OKT3 for CD3ε; 6B10.2 for ζ; TRIM-7 for TRIM; and W6/32 for HLA-1. Texas Red-labelled donkey anti-mouse was used as the secondary antibody.

To further assess the relationship between CD3ε, ζ and TRIM after αβTCR stimulation, we studied the colocalization of ζ/CD3ε and ζ/TRIM in capped cells by means of confocal laser scanning microscopy. Confirming the tight interaction between the two molecules within the αβTCR complex, ζ and CD3ε showed an almost 100% co-localization after 1 hour of T cell activation (Figure 5B, left panel). In marked contrast, under the same experimental conditions, we only observed a partial colocalization between TRIM and ζ (right panel). This finding suggested that the intracellular fate and processing of TRIM differs from that of the other αβTCR components. This assumption was further substantiated by the finding that TRIM did not colocalize with the lysosomal marker Lamp1 at early or late time points of T cell activation (Figure 6A). Furthermore, while the fluorescence signals for both CD3ε and ζ were almost completely lost after prolonged stimulation (due to lysosomal degradation), significant amounts of TRIM were still detectable even after 18 hours of T cell activation (Figure 6B). Thus, despite its association with the αβTCR complex, upon T cell activation the intracellular processing of TRIM differs from that of the other αβTCR components.

## Discussion

The stoichiometry of the TCR complex has been extensively studied [4]. However, the TRIM protein, known to associate with the TCR, was not included in these studies. Using the NAMOS assay [26], we show that in digitonin two αβTCR complexes exist with the stoichiometries TCRαβ CD3ε_2_γδζ_2 _and TCRαβ CD3ε_2_γδζ_2_TRIM_2_. Thus, two TRIM molecules associate with some αβTCR complexes. This is in line with the dimeric nature of TRIM [16].

In digitonin detergent lysates, most αβTCRs did not include TRIM. On one hand, this could indicate that most αβTCRs lack TRIM on the cell surface. This is possible, since TRIM is not required for surface expression of the αβTCR [19-22], but still might be included when present. On the other hand, TRIM might be part of all αβTCR complexes in T cells, but partially dissociates under our detergent conditions. The TCRαβ CD3ε_2_γδζ_2 _stoichiometry is held together by potentially charged amino acids in the transmembrane regions of its subunits [7]. TRIM does not have such amino acids in its transmembrane region [16] and thus might associate less stably with the rest of the αβTCR. Indeed, our data suggest that TRIM is loosely associated with the αβTCR and might be partially (or completely) lost upon purification of the complex.

We have previously shown (and confirmed here) that TRIM co-caps and co-modulates with the αβTCR. Our early studies demonstrated that the reduction of TRIM expression in TCR-modulated T cells was less severe than the reduction of CD3ε and ζ expression. Here, we have extended these observations by showing that the intracellular fate of TRIM differs from that of the other αβTCR components. Indeed, upon prolonged mAb-mediated stimulation of αβ T cells, CD3ε and ζ were no longer detectable by microscopy, most likely due to their degradation within the lysosomal compartment [31-34]. Moreover, confocal laser scanning microscopy revealed an almost complete colocalization of CD3ε and ζ in capped T cells. In marked contrast, a substantial fraction of TRIM did not colocalize with ζ in these cells. Even after 18 hours of stimulation, a substantial fraction of intracellular TRIM was still detectable within T cells and thus was not degraded. These findings were further corroborated by the observation that upon T cell activation TRIM is not targeted to lysosomes (as evidenced by lack of colocalization with the lysosomal marker Lamp1).

The CD3, ζ and TRIM molecules contain the YxxL/I internalization signal [35] that might target proteins for lysosomal degradation [31]. However, not all YxxL/I containing proteins are degraded. In case of the αβTCR it was shown that the Casitas B-lineage lymphoma proteins c-Cbl and Cbl-b (RING-type E3 ubiquitin ligases) together with Cbl adaptor proteins Src-like adaptor protein SLAP and ζ-associated protein ZAP70, target internalized ζ chains to degradation by lysosomes [36-39]. Thus, they are involved in αβTCR downmodulation by inhibiting recycling of internalized αβTCRs. LAPTM5, a lysosomal protein, is also involved in this degradation pathway, since it binds to ubiquitinylated ζ, but not to CD3ε, CD3γ or CD3δ [34]. Our observation that TRIM does not co-localize with ζ and is not degraded might be because TRIM is not bound by SLAP, Cbl, ZAP70 or LAPTM5. Indeed. TRIM does not contain any ITAM to which ZAP70 binds.

The double leucine (LL) internalization motif in CD3γ and CD3δ, but not in ζ, CD3ε or TRIM, does not target for degradation [32,40]. Thus, these signals cannot be the reason for differential targeting of TRIM compared to CD3 and ζ. However, the transmembrane regions, containing sorting motifs in the case of CD3ε and ζ [41,42], are not conserved between CD3/ζ and TRIM. This region of CD3 and ζ is negatively charged, but neutral in TRIM. Thus, this might be the reason for their differential fates after internalization. In conclusion, while TRIM is a component of large αβTCR complexes on the T cell surface, it is differentially processed after T cell activation.

TRIM binds to the ζ subunit of the αβTCR [18]. Since the γδTCR also contains ζ, we tested whether TRIM might associate with the γδTCR and thereby give rise to a second larger detectable γδTCR. This was indeed the case as shown by co-immunopurifications and BN-PAGE analysis. The small human γδTCR complex has a stoichiometry of TCRγδ CD3ε_2_γδζ_2 _[2,28]; the larger complex likely has a TCRγδ CD3ε_2_γδζ_2_TRIM_2 _stoichiometry. At the double negative stage of T cell development, TRIM is already expressed together with the pre-TCR [30], which also includes ζ. Therefore, it is likely that TRIM also associates with the pre-TCR complex. It remains to be seen whether TRIM processing in the context of pre-TCR and γδTCR activation is similar to that observed with the αβTCR complex.

Finally, we present an extension of the NAMOS-assay to 2D BN-/BN-PAGE. The multiprotein complexes of interest are first separated by standard BN-PAGE. Then an antibody to a certain subunit (X) is added and a 2D BN-PAGE is applied. Thus, each individual complex is separately shifted due to the binding of the antibody to X in the second dimension. From the spot patterns of the 2D gel, the number of X molecules per complex can be deduced separately for each complex. Using this method, we show that two CD3ε molecules are present in both the small and the large αβTCR complex. We tested several protocols and determined how to best add the antibody of interest to the TCR complex present within the 1D BN-PAGE (see Methods). A NAMOS-assay 2D BN-/BN-PAGE extends the applicability of the assay to include multiprotein complexes in which a protein exists in more than one complex.

## Conclusions

Here we describe four main results: 1. A novel method to study stoichiometries in 2D BN-/BN-PAGE, in order to define stoichiometries of multiprotein complexes even if they are present in a mixture; 2. The αβTCR complex is found in two forms: TCRαβ CD3ε_2_γδζ_2_TRIM_2 _and TCRαβ CD3ε_2_γδζ_2_, not including TRIM; 3. After stimulation, the αβTCR complex is internalized. CD3 subunits are then degraded in lysosomes, however, TRIM is not subject to this trafficking route and remains intact; 4. TRIM is also associated with the γδTCR.

## Methods

### Cells

Jurkat, Peer and 31.13scTCRβ T cells were maintained in RPMI 1640 (GIBCO BRL) medium supplemented with 5% FCS, as previously described [25]. Peripheral blood T lymphocytes were prepared by E-rosetting from freshly drawn venous blood of healthy donors as described elsewhere [43]. Mouse thymocytes were prepared as described [44]. The human γδ T cell clone was generated from blood as before [28] and was named PF55. PF55 expresses a Vγ9Vδ2 TCR (data not shown).

### Antibodies

The following monoclonal and polyclonal antibodies (Abs) were used for immunoprecipitation, confocal laser scan microscopy, Western blotting, and indirect immunofluorescence as indicated: HLA-I (W6/32, for flow cytometry analysis and cap formation); anti-ζ monoclonal antibody (mAb), clone 6B10.2 (Santa Cruz Biotechnology, Inc., for confocal laser scan analysis and immunoprecipitation); CD3ε mAb OKT3 (for co-capping and flow cytometry analysis); CD3ε clone 2Ad2a2 (IgM; donated by Dr. E. Reinherz, Dana Farber Cancer Center, Boston, MA, for modulation experiments). The rabbit anti-ζ antiserum 448 has been described previously [45]. The anti-TRIM mouse mAb TRIM-7 has also been previously described [18]. Affinity-purified polyclonal anti-TRIM antiserum was prepared as described elsewhere [16,18] and used at 5-10 μg/ml for confocal laser scan analysis.

### Cell lysates and protein purification

To purify the TCR for subsequent BN-PAGE or NAMOS assays, 10^7 ^Jurkat or Peer T cells were incubated in 200 μM pervanadate (8.8 μl 23 mM sodium orthovanadate plus 1.2 μl 30% H_2_O_2_) at 37°C for 5 min in 1 ml RPMI medium to induce tyrosine phosphorylation of the TCR. Cells were lysed in 1 ml lysis buffer (20 mM TrisHCl pH 8, 137 mM NaCl, 2 mM EDTA, 10% glycerol, 10 μg/ml leupeptin, 10 μg/ml aprotinin, 1 mM PMSF, 500 μM Na_3_VO_4_, 1 mM NaF and 1% digitonin). Phosphorylated proteins were purified by incubation with 1 μg of the anti-phosphotyrosine Ab 4G10 (Upstate Biotechnology) and 3 μl protein G-Sepharose (Pharmacia) for minimum 6 hours. The beads were washed twice in lysis buffer, once in BN buffer (0.5 M 6-aminohexanoic acid, 12.5 mM NaCl, 10% glycerol, 2 mM EDTA, 0.5% digitonin, 20 mM BisTris pH7.0, 1 mM PMSF) and eluted in 100 μl BN elution buffer (BN buffer including 50 mM phenylphosphate and 1 unit alkaline phosphatase to dephosphorylate the receptor) for a minimum of 30 min on ice. The scTCR was purified from cell lysates using NP (3-nitro-4-hydroxy-phenylacetate)-conjugated Sepharose, washed three times with BN buffer, and the scTCR eluted in BN-buffer containing 0.5 mM free NIP (NIP-caproic acid, Biosearch Technologies). The eluate was then directly loaded onto a BN gel, or first incubated with Abs against specific subunits, as detailed below.

### Membranes were prepared as described [12]

For immunoprecipitation of αβ or γδTCRs in figure 4, Jurkat or Peer cells were lysed in 1 ml lysis buffer (as above, but containing 0.5% Brij-96 instead of digitonin) and purified by incubation with antibodies against CD3ε (UCHT1, kind gift of Dr. P Beverley, The Edward Jenner Institute for Vaccine Research, Compton, UK), γδTCR (5A6. E9, Pierce Chemical Co.), TCRβ (BV8, Pharmingen), TRIM or ζ(448, described above) coupled to protein G-Sepharose for 4 hours. Beads were washed 4 times with lysis buffer, resuspended in non-reducing sample buffer, boiled 5 min and loaded onto SDS-PAGE.

### BN-PAGE, SDS-PAGE and immunoblotting

BN-PAGE gradient gels (4%-9%) were prepared using a gradient mixer and the Protean II system from BioRad; the stacking gel was 3.2% polyacrylamide. Gel composition, pouring procedure and running conditions have been described [46-49]. In short, the loaded gels were run at 4°C at 20 V overnight. For running BN-PAGE, sample buffer was omitted, but samples in BN elution buffer were carefully overlaid with blue cathode buffer instead (15 mM BisTris pH7.0, 50 mM Tricine and 0.02% Coomassie blue G250). The marker protein was ferritin in its 24-, 48- and 72 meric forms (f1: 440 kDa, f2: 880 kDa, f3: 1320 kDa, respectively). Western blotting was done using PVDF membranes and a semi-dry transfer system, at 18 V for 15 to 20 minutes, with transfer buffer containing 0.1% SDS. SDS-PAGE was performed using standard protocols. The membranes were developed using the Abs mentioned above and secondary Abs were anti-rabbit IgG coupled to HRPO or goat anti-mouse IgG HRPO from Southern Biotechnology. Visualization was performed using the ECL system.

The NAMOS assay was done as described [26,27]. In brief, the indicated amounts of Abs were added to the samples (10 to 20 μl), mixed and incubated for 5-30 min on ice before loading them on BN-PAGE. MPC-antibody conjugates migrate slower in native gels, such that the shift directly reflects the number of Ab molecules bound to the complex.

### NAMOS (Native antibody-based mobility shift) assay in the second dimension

Protein complexes are initially separated by a first dimension (1D) BN-PAGE. They are then brought into contact with anti-subunit Abs while inside the gel. Finally, the protein complex-antibody supercomplexes are separated by a second dimension (2D) BN-PAGE. We tested several experimental procedures for the formation of supercomplexes. The most successful procedure was to load given amounts of Ab in a small volume (e.g. 10 - 20 μl) into the large pocket of the 2D stacking gel. Using the BioRad minigel equipment, electrophoresis is carried out for a few minutes at 100 V, in order to let the Ab enter 1 - 2 mm into the stacking gel. Then the gel run is stopped and the 1D gel lane placed into the large pocket, after which the gel run is continued. During the stacking process, the Ab comes into contact with the protein complexes and forms supercomplexes that are then separated by the 2D separating gel.

### Co-capping Experiments

Resting peripheral blood T cells were washed twice with RPMI (without FCS) and adjusted to 7× 10^6 ^cells/ml in RPMI 1640. 10 μl of this suspension was pipetted onto each reaction field of an adhesion slide (Bio-Rad Laboratories) and allowed to adhere for 10 min at room temperature. Adherent cells were washed twice with RPMI and the slide was blocked for 15 min with complete culture medium. Cap formation was subsequently induced by incubating the cells for 15 min with 15 μl mAb solution (10 μg/ml in complete culture medium), followed by two washes with RPMI 1640 (50 μl per field) and cross-linking of the primary Abs with 15 μl Texas red-labeled donkey anti-mouse antiserum (Dianova; diluted 1:50 vol/vol in culture medium) for 35 min at room temperature in the dark. Capped cells were washed twice with 50 μl PBS supplemented with 0.2% NaN_3 _and then fixed for 3 min with cold methanol (stored at -20°C). The methanol was removed and the cells were fixed for an additional 5 s with ice-cold acetone. Fixed cells were washed twice with PBS and blocked again for 10 min with PBS/1% BSA at room temperature. Subsequently, affinity-purified polyclonal Abs directed at TRIM and CD3ε were added (15 μl per field, concentration 10 μg/ml) and incubated for 30 min at room temperature in the dark. The Ab-labeled cells were then washed once with PBS/BSA (40 μl per field) and three times with PBS. 15 μl secondary Cy2-labeled donkey anti-rabbit Ab (Dianova) were then added (1:125 vol/vol in PBS/1% BSA) and incubated for 30 min at room temperature in the dark. Cells were washed once with PBS/BSA, three times with PBS, and finally at least 5 min with distilled water. The slides were then covered with a cover slip, which was fixed with Elvanol (Calbiochem) for at least 16 h.

Slides were viewed on a Laser scan microscope (Ernst Leitz GmbH) using 570 nm (red emission) and 508 nm (green emission) filters, respectively. In general, the magnification was 40×.

## Competing interests

The authors declare that they have no competing interests.

## Authors' contributions

MS carried out most of the biochemical characterization of the TRIM-containing αβTCRs and drafted the manuscript. GMS performed immunoprecipitation studies and helped to draft the manuscript. GJF did BN-PAGE with the γδTCR. EM and EPD performed biochemical experiments on the αβTCR. PF generated and cultivated the γδ T cell clone. BS performed all microscopy experiments and helped to draft the manuscript. WWAS conceived of the study, and participated in its design and coordination and helped to draft the manuscript. All authors read and approved the final manuscript.
